# Diagnostic accuracy of tru-cut biopsy and acid cytology from patients operated with suspicious for ovarian cancer^**^

**DOI:** 10.1590/1806-9282.20240869

**Published:** 2025-05-02

**Authors:** Fatma Basak Tanoglu, Gurkan Kiran, Caglar Cetin, Burcu Gul, Ozge Pasin, Temel Fatih Yilmaz

**Affiliations:** 1 Bezmialem University, Faculty of Medicine, Department of Obstetrics and Gynecology – İstanbul, Turkey.; 2 Bezmialem University, Faculty of Medicine, Department of Pathology – İstanbul, Turkey.; 3 Bezmialem University, Faculty of Medicine, Department of Biostatistics – İstanbul, Turkey.; 4 Bezmialem University, Faculty of Medicine, Department of Interventional Radiology – İstanbul, Turkey.

**Keywords:** Ultrasound, Biopsy, Cytology

## Abstract

**OBJECTIVE:**

In women who are believed to have ovarian cancer but have a poor performance status or have advanced disease thought to be beyond the scope of primary cytoreductive surgery, neoadjuvant chemotherapy can be administered with acid cytology and/or tru-cut biopsy referral. The aim of this study was to determine the accuracy, adequacy, safety, and reliability of these minimally invasive interventional procedures.

**METHODS:**

This is a retrospective analysis of 63 patients with a suspicion of ovarian cancer who reported to Bezmialem University Hospital between 2014 and 2021, underwent ultrasound-guided acid cytology and tru-cut biopsy, and also had postoperative final pathology results.

**RESULTS:**

On comparing acid cytology and tru-cut biopsy at the same time with the postoperative final pathology results, it was seen that the positive predictive value was 100% in all groups. It was revealed that the sensitivity of acid cytology was 64%, the specificity was 100%, the negative predictive value was 12%, and the accuracy of the test was 65%. The sensitivity of the tru-cut biopsy was 91%, the specificity was 100%, the negative predictive value was 42%, and the accuracy of the test was 92%. In the case of both procedures, the sensitivity was calculated as 93% and the accuracy of the test was calculated as 93%. There were no false-positive cytology and biopsy results.

**CONCLUSION:**

Due to its high reliability and accuracy, the combined application of these minimally invasive methods has the potential to routinely replace more invasive methods for adequate tumor sampling, such as diagnostic laparoscopy or exploratory laparotomy.

## INTRODUCTION

Ovarian cancer has the second highest mortality rate of all gynecological malignancies^1^. Patients do not apply to the hospital at an early stage due to their non-specific symptoms, so only 30% of cases can be diagnosed at stage I or II, and the majority of ovarian cancer cases are diagnosed at an advanced stage^2^. It is a disease with a poor prognosis since it can be diagnosed at an advanced stage, with high recurrence rates despite treatment and low disease-free and overall survival rates in the advanced stage.

The primary treatment for advanced-stage ovarian cancer is primary debulking surgery (PDS) or interval debulking surgery (IDS) after neoadjuvant chemotherapy (NACT), depending on whether surgical excision is possible or not and the comorbidities of the patients^3^. One of the most important factors affecting survival in ovarian cancer is the ability to achieve complete resection or at least optimal cytoreduction (the largest tumor diameter should be less than 1 cm) with surgery^4^. The PDS approach minimizes the tumor burden of patients and contributes effectively to the postoperative chemotherapy process. However, patients with diffuse deep involvement of the small intestinal mesentery, diffuse infiltration of the stomach or duodenum, multiple liver metastases (multisegmental), diffuse carcinomatosis of the small intestine, involvement of the head or the large part of the pancreas, unresectable lymph node disease (e.g., thoracic), multiple lung metastases, and brain metastases are not suitable for PDS^5^. Additionally, IDS is preferred in some patients because their poor preoperative performance and poor nutritional status are associated with postoperative morbidity and mortality^6^.

Today, image-guided biopsy/ascites cytology application using ultrasonography (USG), computed tomography (CT), or magnetic resonance (MR) guidance is precious in the presence of an advanced-stage ovarian cancer suspicion. However, ultrasound-guided methods, with the flexibility to use transvaginal, transabdominal, and transrectal approaches, offer benefits and have minimal risk of complications, high accessibility, and low costs compared to CT or MR guidance^7^. Usually, a histological diagnosis can be achieved without diagnostic laparoscopy or laparotomy by providing a site-specific primary tumor diagnosis. Tissue sampling by diagnostic laparoscopy or laparotomy requires general anesthesia and hospitalization, resulting in higher costs and potential surgical morbidity.

The present study was designed to compare the tru-cut biopsy/ascites cytology samples obtained under preoperative ultrasound guidance and the postoperative final pathology results of the patients operated on with a preliminary diagnosis of ovarian cancer. The primary aim was to compare the consistency of tru-cut biopsy and ascites cytology results with the final pathology results (accuracy). Our secondary aim was to determine these procedures’ safety, reliability, and proficiency.

## METHODS

This study was a retrospective analysis of the tru-cut biopsy, ascites cytology, and postoperative final pathology results of patients with suspicious ovarian cancer who reported to the Department of Obstetrics and Gynecology in Bezmialem University Hospital, Turkey, between January 2014 and December 2021. The Non-interventional Ethics Committee decision number of the study was 2022/31. Data were collected by reviewing electronic patient records, including USG/MR/CT/positron emission tomography reports, laboratory tests, and pathology/surgery reports. The radiologists obtained written informed consent from all patients before the tru-cut biopsy or ascites cytology procedure.

The study included patients who were initially evaluated for ovarian cancer based on imaging, laboratory tests and physical examination findings, but in whom optimal cytoreduction was not possible; patients with a history of previous malignancy and suspected recurrence; patients who were not suitable for PDS due to comorbidities and performance status; and patients who underwent tru-cut biopsy and/or ascitic cytology to make the differential diagnosis of benign (tuberculosis, chronic pelvic infections) and malignant (lymphoma, gastrointestinal system tumors) diseases that were clinicoradiologically similar to ovarian cancer. These patients were also patients with final pathology results who had been operated on with or without NACT. Patients for whom tru-cut/acid cytology results were not available, who died during the NACT treatment, who were lost to follow-up after tru-cut/acid cytology samples were taken or after receiving NACT, and who were under the age of 18 years were excluded from the study.

The patients’ demographic characteristics, physical examination findings, laboratory test results, imaging findings, the site of the biopsy obtained, histopathological features in the tru-cut biopsy and ascites cytology, and final pathology reports were analyzed using the hospital database. The statistical evaluation was performed accordingly.

### Ascites cytology technique

Following the determination of the localization where the procedure would be performed by abdominal USG, immediately after the application of local anesthesia to this area with Locanest Spray containing 10% lidocaine, paracentesis guided by abdominal USG was performed with a 20-Gauge Spinal Needle Quincke 6" to collect at least 60 cc of the ascites fluid.

### Tru-cut biopsy technique

The biopsy was not performed if international normalized ratio (INR) >1.5 or platelet<50,000 cells/mL in the peripheral blood of the patients. In patients using anticoagulants, the drug was discontinued 5 days before the procedure and was restarted after the procedure. Following the determination of the localization where the procedure would be performed with abdominal USG, 5 min after the application of local anesthesia to this area with 3 cc subcutaneous injection of 2% prilocaine hydrochloride, a tru-cut biopsy guided by abdominal USG was performed with the help of 17-Gauge TruGuide Bard coaxial needle and 18-Gauge Max-Core gun to obtain at least two tissue cylinders of 20 mm long.

The Doppler imaging feature of USG was also utilized during the procedure. In this way, the inferior epigastric artery and vein were visualized and vascular injuries were avoided during the needle entry into the abdomen. In addition, attention was paid to the mesenteric vascular structures that may be close to the tissues to be biopsied in the abdomen. Since the vascularity in the tissues can also be evaluated with the help of Doppler imaging, instead of necrotic tissues, live tissues with vascularity were detected in the tumoral areas to be biopsied, and the adequacy of the material that can provide tissue diagnosis was ensured. Another advantage of USG is that it is mobile during the procedure, thus enabling simultaneous tissue acquisition from different localizations within the same mass by moving with the needle.

In this study, radiologists who performed USG-guided paracentesis and tru-cut biopsy procedures have 10, 20, and 25 years of experience, respectively. As indicated in previous studies, we know that performing these procedures with the help of experienced radiologists, especially under USG guidance, has a crucial role in tissue sufficiency^8^.

### Histopathological evaluation

All cytology and tissue samples obtained in our study were examined by two experienced pathologists. All tru-cut biopsy and acid cytology materials examined by the pathologist; reported detailing tumor origin and histological subtype.

### Statistical evaluation

In this study, the descriptive statistics of the qualitative variables were presented as numbers and percentages, and the descriptive statistics of the quantitative variables were presented as mean, median, standard deviation, and minimum and maximum values. Sensitivity, specificity, accuracy, positive predictive value (PPV), and negative predictive value (NPV) were calculated with confidence intervals for evaluating ascites cytology and tru-cut biopsy techniques in terms of final pathology results. The SPSS (version 28) package program was used in the analyses.

## RESULTS

**Figure 1:**
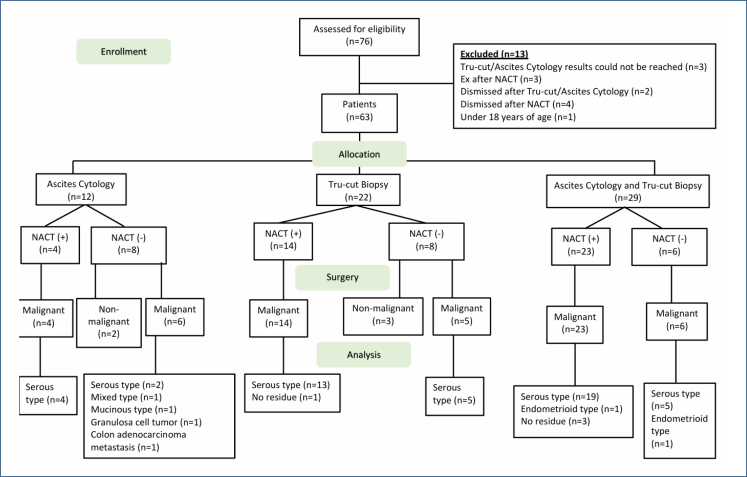
Flowchart of the study.

Between January 2014 and December 2021, a total of 76 patients were evaluated at our department. In the examinations, three patients who were known to have had IDS but whose tru-cut/acid cytology results were not available before NACT, three patients who died during NACT treatment and therefore debulking surgery could not be performed, two patients who were lost to follow-up after tru-cut/acid cytology procedures, four patients whose surgery was not performed in our hospital after NACT, and one patient under the age of 18 years were identified. Sixty-three patients were included in the study after exclusion (Figure 1). The patients were divided into three groups. In 12 of these patients, only acid cytology was obtained before the operation, and only tru-cut biopsy was taken in 22 of them. In 29 patients, both acid cytology and tru-cut biopsy were taken.

The mean age of the 63 female patients included in the study was 62 years, the mean body mass index was 28.5 kg/m², and the mean serum CA125 value was 1,546 U/mL. The complaints, comorbidities, presence of preoperative ascites, Eastern Cooperative Oncology Group scores, clinical stages, and biopsy sites of the patients are presented in Table 1. There were no major differences in the preoperative evaluation between the groups. Tru-cut biopsy was obtained from the omentum in 15 patients, the peritoneum in 24, the pelvic mass in 8, the liver in 2, right cervical lymphadenopathy (LAP) in 1, and left inguinal LAP in 1 patient.

**Table 1:** Preoperative evaluation of patients and biopsied sites.

Parameters (n=63)		
Age (years); mean±SD** (range)		62.5 ± 11.2 (32–81)
BMI^*^ (kg/m²); mean±SD (range)		28.6 ± 5.1 (16.2–44.1)
CA125 (U/ml); mean±SD (range)		1546.0 ± 2365.0 (7.2–2230.0)
Complaint; n%		
	Pain, swelling, palpable mass	42 (66.7)
	Constipation, diarrhea, weight loss	3 (4.8)
	Urinary complaint, abnormal uterine bleeding	6 (9.5)
	Loss of appetite	7 (11.1)
	Shortness of breath	5 (7.9)
Presence of comorbidity***;n%		38 (60.3)
Presence of preoperative ascites; n%		56 (88.9)
ECOG**** score; n%		
	0	10 (15.9)
	1	27 (42.9)
	2	21 (33.3)
	3	5 (7.9)
Clinical stage; n%		
	2	8 (12.7)
	3	38 (60.3)
	4	17 (27)
Biopsy location; n%		
	Omentum	15 (23.9)
	Peritoneum	24 (38.1)
	Mass	8 (12.7)
	Other	4 (6.3)

* Body mass index;

** standard deviation.

*** Comorbidity defined as hypertension, diabetes mellitus, and cardiac, metabolic, and cerebrovascular disorders.

**** Eastern Cooperative Oncology Group. SD: standard deviation; BMI: body mass index; Eastern Cooperative Oncology Group.

Ascites cytology was obtained in 41 of the 63 patients included in the study. In 25 of these patients, both the ascites cytology and final pathology results were reported as "malignant" (true-positive cytology). In two patients, both ascites cytology and final pathology results were reported as "benign" (true-negative cytology). Incompatibility was present in 14 patients. The final pathology results of these patients whose ascites cytology was "non-malignant" were reported as "malignant" (false-negative cytology). There was no patient whose ascites cytology result was "malignant" but whose final result was reported as "non-malignant" (false-positive cytology). Tru-cut biopsy was taken in 51 patients. The biopsy was true positive in 44 of the 51 patients and true negative in three patients. There was incompatibility in four patients (false-negative biopsy). There was no false-positive biopsy. Both ascites cytology and tru-cut biopsy were taken in 29 patients. If at least one of the two procedures was reported as "malignant," the result was evaluated as "malignant." There was a true-positive result in 27 patients and a false-negative result in two patients. In two of the true-positive patients, tru-cut biopsy was benign, while acid cytology was reported as malignant, and in eight of them, tru-cut biopsy was malignant, while acid cytology was reported as benign. True negativity could not be assessed since no patients in this group had a "non-malignant" final result. False positivity was not present. Among the patients included in the study, the final pathology results of five patients were reported as benign. These patients were thought to have ovarian cancer clinically and radiologically.

When the pathology results of patients with acid cytology and tru-cut biopsy were compared with the postoperative final pathology results, we found that the PPV was 100% in all groups. We found that the sensitivity of the acid cytology procedure was 64%, the specificity was 100%, the NPV was 12%, and the accuracy of the test was 65%. We found that the sensitivity of the tru-cut biopsy procedure was 91%, the specificity was 100%, the NPV was 42%, and the accuracy of the test was 92%. In the case of both procedures, we found that the sensitivity was 93% and the accuracy of the test was 93%. However, the specificity and NPV could not be calculated since there were no patients in the third group whose final pathology result was reported as benign (Table 2).

**Table 2:** Evaluation of the sensitivity, specificity, positive predictive value*, negative predictive value**, and accuracy of ascites cytology and trucut biopsy.

Procedures	Sensitivity (95%CI) (%)	Specificity (95%CI) (%)	PPV (95%CI) (%)	NPV (95%CI) (%)	Accuracy (%)
Ascites cytology (n=41)	64.1 (47.1–78.3)	100 (19.7–100)	100 (83.4–100)	12.5 (2.2–39.5)	65.9
Tru-cut biopsy (n=51)	91.7 (79.1–97.2)	100 (30.9–100)	100 (89.9–100)	42.8 (11.8–79.7)	92.2
Ascites cytology+tru-cut biopsy (n=29)	93.1 (75.7–98.7)	N/A	100 (84.4–100)	N/A	93.1

* Positive predictive value.

** Negative predictive value. Patients who underwent both procedures were included in the first and second groups.

CI: confidence interval; PPV: positive predictive value; NPV: negative predictive value.

The present study performed 97 ultrasound-guided minimally invasive procedures. The tru-cut biopsy material was reported as insufficient in one patient and acid cytology material in four patients, but sufficient tissues and cells were obtained in the second attempt. Therefore, the results of 92 procedures were available, 51 tru-cut biopsy and 41 acid cytology. Adverse events occurred in two of them during the procedure. One was subcutaneous hemorrhage after tru-cut biopsy, and the other was drainage catheter-related closed perforation in the colon. The adverse event rate was 2%.

## DISCUSSION

In recent years, IDS has become an option for advanced ovarian cancer. Randomized controlled studies on large populations have revealed that IDS has the advantages of achieving optimal cytoreduction, providing palliative chemotherapy, and increasing the quality of life when evaluated together with emotional and cognitive functions. It also saves time in the preparation of the operation for patients who are not suitable for the operation because of the high risk of morbidity/mortality after cytoreductive surgery^9,10^. Tissue diagnosis should usually be obtained by image-guided paracentesis, biopsy, or surgery (laparoscopy, laparotomy) before NACT can be initiated. Ascites cytology and tru-cut biopsy techniques are less invasive methods for diagnosing malignancies. These techniques can be particularly useful in patients unsuitable for primary surgery and may obviate the need for open or closed surgical procedures.

The accuracy of ascites cytology and tru-cut biopsy was evaluated in the present study. In the study by Baransi et al., including 551 patients, the sensitivity, specificity, PPV, and NPV values of ascites cytology in the diagnosis of epithelial ovarian cancer were 80.6, 100, 100, and 16.7%, respectively^11^. However, we found that the sensitivity, specificity, PPV, NPV, and accuracy of acid cytology were 64, 100, 100, 12, and 65%, respectively. In our study, patients who underwent acid cytology also had non-epithelial ovarian cancer and metastases from another cancer in their final pathology results. Serous adenocarcinomas may show papillary configuration and psammoma bodies on cytological evaluation, whereas their absence in other acid-produced carcinomas may explain the low sensitivity.

In the study by Zikan et al., in which 195 tru-cut biopsies including 190 patients were performed, the diagnostic accuracy was 98.3%^12^. In our study, the diagnostic accuracy of tru-cut biopsy was found to be 92%. The only study similar to ours was the study published by Vlasak et al. in 2020, which compared the diagnostic reliability, accuracy, and safety of ultrasound-guided ascites cytology and tru-cut biopsy, covering 79 patients. The rates of the confirmation of malignancy and compliance with the final report were, respectively, 72.9 and 43.7% for the cytology and 95.8 and 95.4%, respectively, for the tru-cut biopsy. However, not all patients included in the study had final pathology results. There are also patients whose disease progresses and cannot be operated on despite NACT^13^.

Mascilini et al. evaluated the diagnostic accuracy of transvaginal ultrasound-guided biopsy. The accuracy of biopsy over surgery was found to be 94% (96/102), which was similar to ours, while six false-negative cases (6%) were reported. Biopsy correctly identified 86 primary invasive tubo-ovarian carcinomas and 10 metastatic tumors. In addition, tumor location (prevesical peritoneum) and size (<8 mm) were cited as the major predictive factors for ultrasound-guided biopsy failure (false negativity)^14^. False-positive cytology and biopsy results, which could lead to unnecessary NACT, were not reported in any other study, including ours. These results show that NACT can be administered safely to patients determined to be "malignant" with ascites cytology and tru-cut biopsy to diagnose ovarian cancer. However, it is seen that the sensitivity for tru-cut biopsy is much higher than that for ascites cytology, so a tru-cut biopsy is more reliable than ascites cytology in detecting patients with ovarian cancer.

While the adverse event rate detected in our study was 2%, this rate was similarly found to be 1% in the study by Zikan et al.^12^. In another study evaluating the accuracy, adequacy, safety, and clinical use of tru-cut biopsy in gynecological cancers, infection-related complications occurred in four of 300 patients (1.3%), who underwent biopsy^15^. To achieve these rates, adverse events observed in previously published studies related to tru-cut biopsy were considered, and the procedure was performed by confirming that the patients’ routine platelet and INR values were appropriate before the procedure and attention was paid to sterilization.

The fact that all patients had final pathology results and that we could compare ascites cytology and tru-cut biopsy simultaneously both with the final pathology results and among themselves reflects the study's strength. Being single-center in design and having two pathologists evaluating all pathology reports increase the study's objectivity. The 10 years or more experience of the radiologists who performed these minimally invasive procedures under USG guidance added strength to our research. However, because of the study's retrospective nature, the choice of who would or would not perform the procedures and the decision about suitability for surgery after NACT were variables we could not control. In clinical practice, disagreements are not uncommon within the council that decides on the suitability of these patients for surgery. The relatively small number of patients included in the study also limits the results obtained regarding the safety, adequacy, reliability, and accuracy parameters we investigated in the study.

## CONCLUSION

Ultrasound-guided tru-cut biopsy currently appears to be the single best minimally invasive method for collecting sufficient tissue samples for histopathological examination. It is also suitable for confirming recurrences, diagnosing non-gynecological malignancies infiltrating the pelvic organs, or distinguishing between malignant and benign tumors. When acid cytology can be added to this procedure, the probability of false-negative results appears to be much less. However, when tru-cut biopsy is not possible, considering ascites cytology alone as a diagnostic method does not seem appropriate, as it would not allow a significant proportion of patients suitable for IDS to benefit from NACT. Due to its high reliability and accuracy, the combined application of these minimally invasive methods has the potential to routinely replace more invasive methods for adequate tumor sampling, such as diagnostic laparoscopy or exploratory laparotomy.
